# Depletion of UDP-Glucose and UDP-Galactose Using a Degron System Leads to Growth Cessation of *Leishmania major*

**DOI:** 10.1371/journal.pntd.0004205

**Published:** 2015-11-03

**Authors:** Sebastian Damerow, Carolin Hoppe, Giulia Bandini, Patricia Zarnovican, Falk R. Buettner, Carsten G. K. Lüder, Michael A. J. Ferguson, Françoise H. Routier

**Affiliations:** 1 Department of Cellular Chemistry, Hannover Medical School, Hannover, Germany; 2 Division of Biological Chemistry and Drug Discovery, College of Life Sciences, University of Dundee, Dundee, United Kingdom; 3 Institute for Medical Microbiology, Georg-August University, Goettingen, Germany; Liverpool School of Tropical Medicine, UNITED KINGDOM

## Abstract

Interconversion of UDP-glucose (UDP-Glc) and UDP-galactose (UDP-Gal) by the UDP-Glc 4´-epimerase intimately connects the biosynthesis of these two nucleotide sugars. Their *de novo* biosynthesis involves transformation of glucose-6-phosphate into glucose-1-phosphate by the phosphoglucomutase and subsequent activation into UDP-Glc by the specific UDP-Glc pyrophosphorylase (UGP). Besides UGP, *Leishmania* parasites express an uncommon UDP-sugar pyrophosphorylase (USP) able to activate both galactose-1-phosphate and glucose-1-phosphate *in vitro*. Targeted gene deletion of *UGP* alone was previously shown to principally affect expression of lipophosphoglycan, resulting in a reduced virulence. Since our attempts to delete both *UGP* and *USP* failed, deletion of *UGP* was combined with conditional destabilisation of USP to control the biosynthesis of UDP-Glc and UDP-Gal. Stabilisation of the enzyme produced by a single *USP* allele was sufficient to maintain the steady-state pools of these two nucleotide sugars and preserve almost normal glycoinositolphospholipids galactosylation, but at the apparent expense of lipophosphoglycan biosynthesis. However, under destabilising conditions, the absence of both UGP and USP resulted in depletion of UDP-Glc and UDP-Gal and led to growth cessation and cell death, suggesting that either or both of these metabolites is/are essential.

## Author Summary

Leishmaniases are a set of tropical and sub-tropical diseases caused by protozoan parasites of the genus *Leishmania*. They affect about 12 million people and cause a high morbidity. Since treatments against all forms of leishmaniasis are limited in number and efficacy, many efforts are made to identify potential drug targets and develop new therapies. Although considerable progress in genetic manipulation of *Leishmania* parasites have been made, it remains difficult to study molecules or metabolic pathways essential for parasite viability and growth. In the present work, we used a combination of gene deletion and conditional protein destabilization to demonstrate that biosynthesis of the nucleotide sugar UDP-glucose and its derivative UDP-galactose is essential for parasite growth. Addition of a specific ligand to the culture medium of the engineered parasite protected the targeted enzyme from degradation and enabled cell growth and viability. However, removal of the stabilizing compound led to depletion of UDP-glucose and UDP-galactose, growth arrest and cell death. This work thus opens a new possibility for the study of essential proteins.

## Introduction

Leishmaniases are a set of tropical and sub-tropical diseases caused by protozoan parasites of the genus *Leishmania* and transmitted by the bite of a sandfly. The severity of the diseases depends on parasite species as well as the immune status of the host and ranges from self-healing cutaneous lesions to fatal visceral infections [1]. According to the World Health Organisation, more than 1 million new cases of cutaneous leishmaniasis and at least 30 000 deaths due to visceral leishmaniasis occur annually. Current treatments are far from ideal and the need to develop new treatments against leishmaniasis is generally recognised [2].

Advances in genetic manipulation of *Leishmania* parasites has considerably facilitated the characterisation of metabolic processes and molecules important for parasite proliferation or virulence [3,4]. Nevertheless, the study of essential genes remains difficult since knockouts can only be performed if rescue strategies such as nutrient supplementation or ectopic gene copies can be used. Unfortunately, most *Leishmania* species including *L*. *major* and *L*. *donovani* lack a functional RNAi pathway [4]. More recently, a system for conditional destabilisation of protein has been described. This original system involves fusion of a mutated FK506 binding protein destabilising domain to the protein of interest and its stabilisation by addition of a specific ligand [5]. However, this system has not yet been applied to essential proteins.

The promastigote stage of *Leishmania* parasites synthesises a dense surface glycocalyx and secretes proteophosphoglycans (PPGs) that are both essential for its development and survival within the insect vector [6]. The glycocalyx contains various GPI-anchored molecules including glycoproteins, lipophosphoglycans (LPGs), proteophosphoglycans (PPGs) and the abundant glycoinositolphospholipids (GIPLs) [7,8]. These surface and secreted glycoconjugates are rich in galactose and mannose and thus their biosynthesis requires an abundant supply of GDP-α-D-mannose (GDP-Man) and UDP-α-D-galactose (UDP-Gal). Biosynthesis of GDP-Man is required for formation of the glycocalyx [8] and for biosynthesis of the carbohydrate storage polymer β1,2-mannan [9]. Since these are essential for virulence, the enzymes involved in GDP-mannose biosynthesis are considered as potential drug targets [10–12].

To address the importance of UDP-Gal biosynthesis in *Leishmania*, we previously targeted the UDP-glucose pyrophosphorylase (EC 2.7.7.9) (UGP) [13]. This enzyme catalyses the transformation of α-D-glucose-1-phosphate (Glc-1P) and UTP into UDP-glucose (UDP-Glc) [14]. UDP-Glc can then be interconverted into UDP-Gal by the UDP-Glc 4´-epimerase. Unexpectedly, although reduced, galactosylation of the glycocalyx was not abolished in the UGP deficient mutant [13]. The residual galactosylation was explained by the discovery of an unusual UDP-sugar pyrophosphorylase (USP) (EC 2.7.7.64) that can activate α-D-galactose-1-phosphate (Gal-1P) and Glc-1P with UTP to form the corresponding UDP-sugar [15]. Recently, deletion of *USP* in *L*. *major* was shown to abolish conversion of Gal-1P into UDP-Gal confirming its role in galactose salvage [16]. Interestingly, the hexose transporters of the related trypanosomatids *T*. *brucei* and *T*. *cruzi* are unable to transport galactose [17,18] and the only route to UDP-Gal formation in these two parasites is via epimerisation of UDP-Glc, which is essential for parasite growth [19–21].

In this study, we applied a combination of gene deletion and protein destabilisation to evaluate the importance of the UDP-Glc/UDP-Gal biosynthesis. Thus, biosynthesis of these two nucleotide sugars could be reduced to minimal level, leading to growth cessation and cell death.

## Methods

### Parasite culture

Promastigote cultures of wild type *L*. *major* MHOM/SU/73/5ASKH and respective mutant cell lines were grown at 27°C in standard culture media consisting of M199 medium (Invitrogen) supplemented with 10% heat inactivated fetal calf serum, 40 mM Hepes pH 7.5, 0.1 mM adenine, 0.0005% hemin, 0.0002% biotin. Antibiotics (Invivo Gen) were added as required at a concentration of 5 μg/mL phleomycin, 50 μg/mL Hygromycin, 30 μg/mL puromycin and 100 μg/mL nourseothricin. For the maintenance of the *ugp* ^-/-^ *usp* ^-/c^ mutant described below, 1 μM FK506 (LC laboratories) was added to medium. If lower FK506 concentrations were used, parasites were pelleted and washed twice with medium before being resuspended with the desired FK506 concentration.

### Generation of *L*. *major ugp* ^-/-^ *usp* ^-/c^ strain

Sequence of all primers used can be found in S1 Table. A USP gene (*LmjF*17.1160) replacement cassette carrying the puromycin resistance gene *PAC* was constructed by double-joint PCR. 2.3 kb of the 5’-UTR and 1.3 kb of the 3’-UTR were amplified with the primer pair 5UTR_1fw/5UTR_1rev and 3UTR_1fw/ 3UTR_1rev respectively. The *PAC* gene was amplified with primer pair OL_PACfw/OL_PACrev having 52bp homology to the 5´-UTR (forward primer) or 51bp homology to the 3´-UTR (reverse primer). After fusion of the 3 amplicons, the final cassette was obtained by nested PCR with primer pair 5UTR_3fw/3UTR_3rev and ligated into pYES-NTA vector via Not I restriction sites (plasmid #3612). The deletion cassette was excised with Bbv CI/Xcm I, separated on TAE 0.7% agarose, extracted from gel, ethanol precipitated and dissolved in water at ~ 2 μg/μl. Additionally, a knock-in construct was created based on the generic plasmid pGEM-*MCS1*-*PHLEO*-DST IR-dd*MCS2* (B6323, kind gift of S. M. Beverley) [5]. The phleomycin resistance gene was first exchanged by the nourseothricin resistance gene *SAT* using Msc I and Rsr II restriction sites, yielding pGEM-*MCS1*-SAT-DST IR-dd*MCS2* (plasmid #3625). Then 2.0 kb of the vicinal *USP* 5’UTR were cloned with primer pair SD177/SD178 and inserted upstream of *SAT* in the Nde I and Spe I sites. Similarly, 1.8 kb from the start of the *USP* gene were amplified with primers SD175/USP3rev and inserted directly behind the destabilisation domain (dd) via the Bgl II and Sac II sites (plasmid #3628). The knock-in cassette was excised with Nde I/Sac II and purified.

Transfections were performed by electroporation, using the high voltage protocol and cytomix buffer as previously described [22]. *ugp* ^-/-^ *usp* ^-/+^ (Δ*ugp*::*BLE*/Δ*ugp*::*HYG/*Δ*usp*::*PAC/USP*) clones were generated by transfection of the *USP* gene replacement cassette into the *L*. *major ugp* ^-/-^ mutant (Δ*ugp*::*BLE*/Δ*ugp*::*HYG*) [13]. Likewise *ugp* ^-/-^ *usp* ^-/c^ clones (Δ*ugp*::*HYG*/Δ*ugp*::*BLE*/Δ*usp*::*PAC*/*SAT-FKBP* ^*FK506i*^::*USP*) were obtained by replacing one *USP* allele by the knock-in cassette to create *ugp* ^-/-^ *usp* ^+/c^ clones (Δ*ugp*::*HYG*/Δ*ugp*::*BLE*/*USP*/*SAT-FKBP* ^*FK506i*^::*USP*) and subsequently deleting the remaining USP allele. These clones were recovered on semi-solid plates containing 1% Noble agar, appropriate antibiotics and 1 μMFK506. Analysis of 3 single clones is presented in this manuscript. Correct insertion of the respective gene replacement or knock-in cassette was analysed by PCR and Southern blot. Genomic DNA was isolated from Log phase parasites by phenol/chloroform extraction. Southern blots were performed according to standard methods. DIG-labelled probes were synthesised using the DIG DNA labelling mix from Roche with primer pairs SD1/USP1_rev; SD176/SD21 and SD70/SD71.

### Western blotting

Early Log phase promastigote lysates were separated on SDS-PAGE and transferred onto PVDF membranes. Equal protein load was assessed by Coomassie brilliant blue protein staining of an identically loaded SDS-PAGE ran separately. Infrared detection on Li-Cor Odyssey Imager was performed after incubation with monoclonal anti-LPG WIC79.3 antibody (protein G purified from mouse hybridoma cells) and goat anti-mouse IgG IR800 Dye 800 CW (Li-Cor) at dilutions of 1:1,000 and 1:20,000, respectively. *L*. *major* USP was detected using a 1:20,000 dilution of polyclonal rabbit anti-serum [16] and goat anti-rabbit IgG IR800 Dye 800 CW (Li-Cor).

### *In vitro* enzyme assays

Conversion of Gal-1P into UDP-Gal or Glc-1P into UDP-Glc by Log phase promastigotes lysates was measured as previously described [16]. Statistical analyses were performed with GraphPad Prism 4 software (Graph-Pad software Inc., La Jolla, CA) using one-way ANOVA with Tukey multiple comparison post-test.

### Quantification of nucleotide sugars

Nucleotide sugar pools of wild type *L*. *major* and the *ugp* ^-/-^ *usp* ^-/c^ clones conditionally stabilized (1 μM FK506) and partially destabilized (0.01 μM FK506) were measured by high performance liquid chromatography-electrospray ionisation-tandem mass spectrometry using multiple reaction monitoring as previously described [23]. Each nucleotide sugar peak was integrated, normalized to its internal standard GDP-Glc and to the respective nucleotide sugar standard of known concentration. Medium containing 0.01μM FK506 was preferred for this experiment to reduce cell death. Statistical analyses were performed with GraphPad Prism 4 software using a paired t-test to compare *ugp* ^-/-^ *usp* ^-/c^ grown in high and low FK506 containing medium.

### GIPL analysis by MALDI-TOF MS

Log phase promastigotes were extracted in chloroform/ methanol/ water (5:10:4), purified over a C18/ SepPak Plus column (Waters) and dried under a nitrogen stream as described previously [24]. GIPLs (1.6x10^7^ cell equivalents) were dissolved in CHCl_3_/ MeOH/ H_2_O (15:30:4), mixed with 6-Aza-2-thiothymidine matrix (5 μg/μl H_2_O) and spotted on a metal target plate. Matrix-assisted laser desorption ionization-time of flight (MALDI-TOF) mass spectrometry was carried out with a Voyager DE Pro (Applied Biosystems, Foster City, CA) in negative-ion reflector mode over the m/z range 900–2000 with an accelerating voltage of 20 kV and a delay of 150 ns. Final mass spectra represented an average of 6–8 spectra, each of which is acquired from 200 laser shots. Spectra were processed using Data Explorer Software V4.8 applying “Advanced Baseline Correction” and “Noise Removal”.

### Analysis of cell viability

Late Log phase promastigotes were washed with PBS and transferred to medium containing 1 μM, 0.05 μM, 0.005 μM FK506 or no FK506 at a cell density of 10^6^ cells/ml. After 3 days, parasites were diluted with fresh medium to 6 x 10^5^ cells/ml and allowed to grow for another 2 days. Parasites were then harvested by centrifugation, washed twice in ice-cold PBS, and resuspended at a density of 5 × 10^6^ parasites/ml. After addition of 5 μl of 7-amino-actinomycin D (7-AAD) solution per 100 μl and incubation for 15 min at room temperature in the dark, 8,000 cells of each sample were analysed by FACS (FACSCalibur).

Statistical analyses were performed with GraphPad Prism 4 software using one-way ANOVA with Tukey multiple comparison post-test.

## Results

### Generation of a mutant with minimal UDP-Glc/UDP-Gal biosynthesis

Upon deletion of *UGP*, significant amount of UDP-Glc and UDP-Gal was still synthesized by *Leishmania* parasites [13], which is likely due to a partial compensation by USP [15]. We thus anticipated that deletion of both *USP* and *UGP* would abrogate biosynthesis of these two nucleotide sugars. In a first step, we were able to replace one allele of *USP* with a gene encoding resistance to puromycin (*PAC*) in the previously described *ugp* ^-^ mutant [13] to generate an heterozygous mutant named *ugp* ^-/-^ *usp* ^-/+^ (Δ*ugp*::*BLE*/Δ*ugp*::*HYG/*Δ*usp*::*PAC/USP*). However, despite several attempts, replacement of both alleles of *USP* in the *ugp* ^-^ mutant remained unsuccessful.

**Fig 1:**
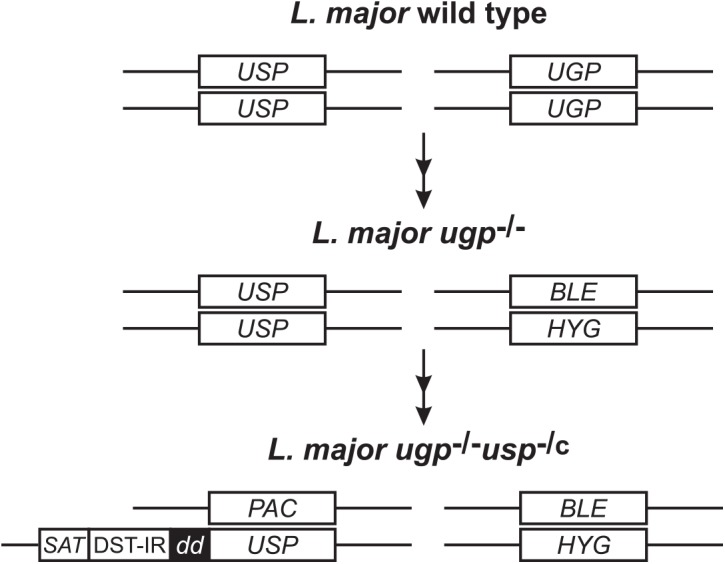
Generation of *Leishmania major ugp* ^-/-^ *usp* ^-/c^ mutant. *L*. *major ugp* ^-/-^ *usp* ^-/c^ mutant was generated from a UGP deficient mutant [11] by replacement of a USP allele with a construct encoding USP N-terminally fused to a mutated FK506 binding protein destabilizing domain (ddUSP). This system enables stabilisation of the fusion protein by addition of FK506 to the culture medium and its proteasomal degradation in absence of FK506. The second USP allele was replaced by transfection with a puromycin resistance gene (*PAC*). *BLE*, phleomycin resistance gene; *HYG*, hygromycin resistance gene; *SAT*, nourseothricin resistance gene.

Aiming at reducing the UDP-Glc/UDP-Gal biosynthesis to a minimum, we replaced one of the *USP* allele by a cassette encoding USP N-terminally fused to a mutated FK506 binding protein (FKBP) destabilizing domain called thereafter ddUSP whereas the second *USP* allele and *UGP* alleles were deleted (Fig 1). The degron system enables stabilisation of the protein of interest by addition of FK506 to the culture medium and its conditional destabilisation by removal of FK506 [5]. The resulting conditional mutant (Δ*ugp*::*HYG*/Δ*ugp*::*BLE*/Δ*usp*::*PAC*/*SAT-FKBP* ^*FK506i*^::*USP*) was analysed by southern blot to confirm correct integration of the deletion and knock-in cassettes (S1 Fig) and was named *ugp* ^-/-^ *usp* ^-/c^. Three independent clones were selected and analysed in this study.

**Fig 2:**
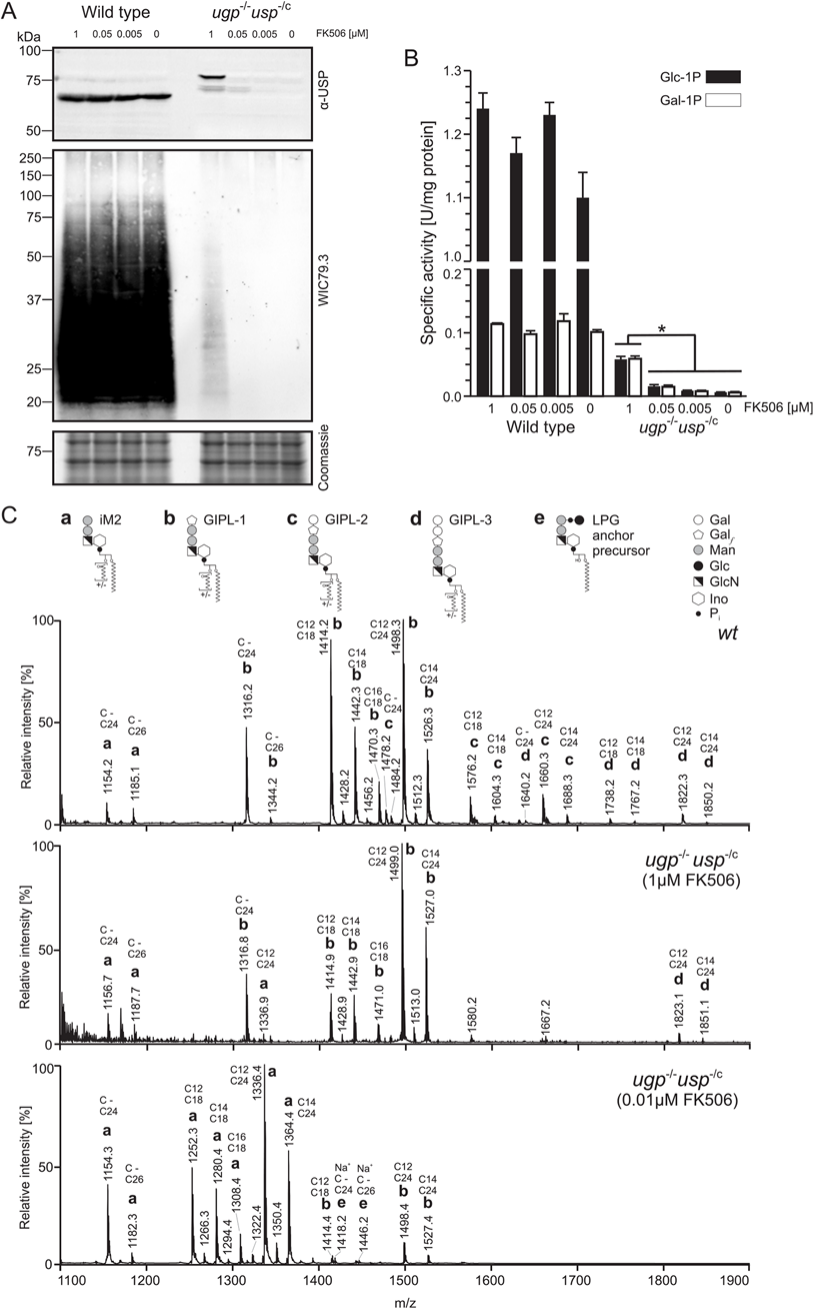
Depletion of USP in *L*. *major ugp* ^-/-^ *usp* ^-/c^ mutant and effect on glycoconjugates biosynthesis. (A) Western blot of Log phase wild type or *ugp* ^-/-^ *usp* ^-/c^ promastigotes lysates probed with an anti-USP antiserum (upper panel) or the anti-LPG monoclonal antibody WIC79.3 (middle panel). Parasites were cultivated in medium containing 1, 0.05, 0.005 µM or no FK506 as indicated. Loading was assessed by Coomassie staining of an identically loaded SDS-Page ran separately (lower panel). (B) *In vitro* conversion of Glc-1P (black bars) or Gal1P (white bars) into nucleotide sugars by cell lysates of wild type and *ugp* ^-/-^ *usp* ^-/c^ mutant cultivated in medium containing various FK506 concentrations as indicated. Values represent the mean ± SEM from n = 3 independent cultures; for the *ugp* ^-/-^ *usp* ^-/c^ mutant, each culture represents a different clone. Significant difference (*p < 0.01), One-way ANOVA with Tukey post test. (C) Negative-ion MALDI spectra of glycosylinositolphospholipids (GIPLs) isolated from wild type and *ugp* ^-/-^ *usp* ^-/c^ mutant grown in presence of 1 or 0.01 μM FK506. Each prominent peak is annotated with its m/z value, the letter a, b, c, d or e referring to the structure depicted above. The lipid moiety consists of a 1-alkyl-2-*lyso*-phosphatidylinositol or 1-alkyl-2-acyl-phosphatidylinositol. The length of the alkyl and acyl chains is indicated above each peak; C- indicates absence of an alkyl chain.

The absence of UGP had been demonstrated in the parental strain by Western blot using a rabbit polyclonal anti-UGP serum [13]. To confirm the functionality of the degradation system in the different *ugp* ^-/-^ *usp* ^-/c^ clones, we analysed the USP protein level in lysates of Log phase promastigotes using a rabbit polyclonal anti-USP serum [16]. As shown in Fig 2A (upper panel), in presence of 1 μM FK506, ddUSP was clearly detectable at approximately 81 kDa (USP: ~69 kDa; destabilising domain: ~12 kDa) whereas in wild type parasites, native USP migrated at approximately 69 kDa. The amount of ddUSP in the different clones stabilised with 1μM FK506 was estimated to 50–60% of wild type from the signal intensity. As anticipated, at lower FK506 concentrations of 0.05 μM, only traces of ddUSP were still discernible and the enzyme was essentially absent if the FK506 concentration was reduced to 0.005 μM (Fig 2A, upper panel, right). As expected USP level was not affected by the FK506 concentration in wild type parasites (Fig 2A, upper panel, left).

### Regulation of the UDP-Glc/UDP-Gal biosynthesis in *L*. *major* using the degron system

The ability of wild type and mutant parasites to convert Glc-1P and Gal-1P into the corresponding nucleotide sugars was measured in promastigote lysates and is presented in Fig 2B. Enzymatic activities measured with lysates of wild type parasites were unaffected by FK506 addition to the growth medium.

When using Glc-1P as substrate, lysate of the *ugp* ^-/-^ *usp* ^-/c^ mutant grown with 1 μM FK506 presented about 4.5% of the activity obtained with wild type lysate. Since the contribution of USP was previously estimated of about 10% in a UGP deficient mutant [13], the present value is consistent with stabilisation of ddUSP produced by one allele. Only 1% activity could still be measured when the enzyme was stabilised with 0.05 μM FK506 and this dropped below the limits of detection when lower drug concentrations were used.

Similar values were obtained when Gal-1P was used as substrate. With 1μM FK506 in the culture medium, the stabilised ddUSP had an activity of 0.06 U/mg that represents approximately 55% of the wild type activity (0.11 U/mg). When grown in presence of 0.05 μM FK506, the *ugp* ^-/-^ *usp* ^-/c^ mutant lysate showed a low UDP-Gal synthesis capacity of 13% of wild type activity. The enzymatic activity became undetectable with lower drug concentrations.

These results provide evidence for the functionality of the conditional degron system, which allows us to reduce USP protein level and enzymatic activity to background level.

### Analysis of LPG and GIPLs

We analysed the effects of regulating UDP-Glc and UDP-Gal biosynthesis, using the degron system, on the production of LPG and GIPLs. Equal amounts of cell lysate were run on 12% SDS-PAGE, blotted on PVDF membrane and developed using the monoclonal antibody WIC79.3, which is specific for galactosylated phosphoglycans repeat units (Fig 2A, middle panel). Loading was controlled by Coomassie blue staining (Figs 2A, lower panel, and S2). As expected, the *ugp* ^-/-^ *usp* ^-/c^ mutant stabilised by the presence of 1 μM FK506 only showed a faint LPG signal, weaker as the signal of the *ugp* ^-/-^ parental strain (S3 Fig). This faint LPG signal (Figs 2A and S3 show the clone with the highest LPG signal) was reduced to below the limits of detection if the mutant was supplemented with lower FK506 concentrations (Fig 2A).

Interestingly, our first attempt to generate an *ugp* ^-/-^ *usp* ^-/c^ mutant resulted in clones devoid of LPG. However, ectopic expression of UGP in these clones or in the *ugp* ^-/-^ *usp* ^-/+^ clone from which they were derived did not restore LPG biosynthesis. This suggests that *Leishmania* can adapt to the low UDP-Glc/UDP-Gal availability by shutting down LPG biosynthesis, which is dispensable for *in vitro* growth [24,25]. In order to avoid *in vitro* adaptation, the clones presented in this study were maintained in 1 μM FK506 and the number of passages limited. Moreover, LPG expression was regularly checked.

GIPLs purified from the *ugp* ^-/-^ *usp* ^-/c^ mutant and wild type parasites were analysed by MALDI-TOF mass spectrometry in negative ion mode (Figs 2C and S3). The observed ions were annotated based on structures previously reported and correspond to type 2 GIPLs with 0, 1, 2 or 3 galactose residues termed iM2, GIPL-1, GIPL-2 and GIPL-3 respectively [26]. Additional heterogeneity arises from the lipid part that is either an alkylacylglycerol or lysoalkylglycerol with aliphatic chains of various lengths (S2 Table). Remarkably, stabilisation of ddUSP with 1 μM FK506 was sufficient to maintain galactosylation of GIPLs (Figs 2C and S3). However, at low FK506 concentration, GIPLs were virtually agalactosylated. Only traces of GIPL-1 (containing one galactofuranose residue) were still visible at a FK506 concentration of 0.01 μM. This loss of galactosylation upon ddUSP destabilisation reflects depletion of the cell’s UDP-Glc and UDP-Gal pools as shown below.

**Fig 3:**
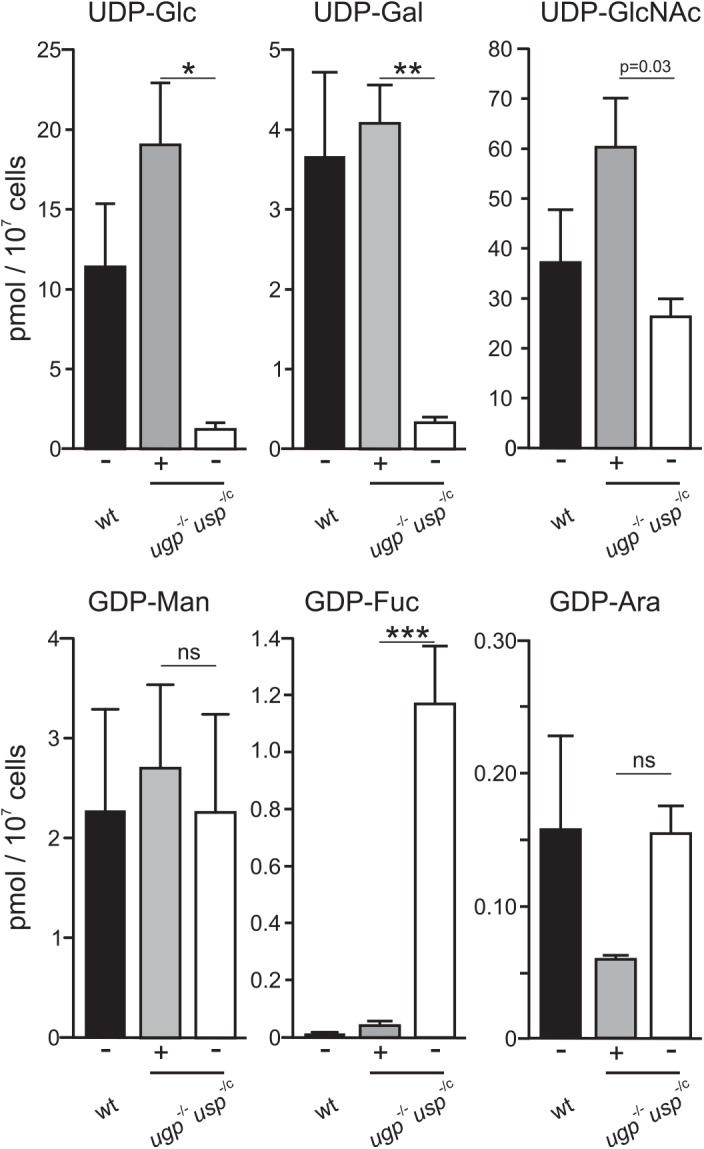
Analysis of nucleotide sugar pools in *L*. *major* wild type and *ugp* ^-/-^ *usp* ^-/c^ mutant. Nucleotide sugars were extracted from mid Log phase wild type (wt) and *ugp* ^-/-^ *usp* ^-/c^ promastigotes grown in presence of 1 μM (+) or 0.01 μM (-) FK506 and measured by liquid chromatography-electrospray ionisation-tandem mass spectrometry with multiple reaction monitoring. Values represent the mean ± SD from n = 3 independent cultures; for the *ugp* ^-/-^ *usp* ^-/c^ mutant, each culture represents a different clone. Significant differences (*p < 0.01, ** p < 0.005, *** p < 0.001), paired t-test.

The nucleotide sugar pools in wild type *L*. *major* and the *ugp* ^-/-^ *usp* ^-/c^ clones were measured by high performance liquid chromatography-electrospray ionisation-tandem mass spectrometry using multiple reactions monitoring as previously described [23]. Surprisingly, stabilisation of the enzyme produced from a single ddUSP allele (with 1 μM FK506) was sufficient to maintain the steady-state pools of UDP-Glc and UDP-Gal (Fig 3). In all clones, a strong reduction of the UDP-Glc and UDP-Gal pools was nevertheless observed upon destabilisation of USP in medium containing 0.01 μM FK506. In contrast, the GDP-Man and GDP-Ara pools did not significantly differ from the pools measured in wild type parasites (Fig 3). Unexpectedly, the pool of UDP-N-acetylglucosamine (UDP-GlcNAc) seemed to slightly decrease in the *ugp* ^-/-^ *usp* ^-/c^ mutant upon USP destabilisation (0.01μM FK506) whereas the GDP-fucose (GDP-Fuc) pool, which is very small in wild type parasite, was strongly elevated in these conditions.

### Analysis of growth and cell death in *L*. *major ugp* ^-/-^ *usp* ^-/c^ mutant

**Fig 4:**
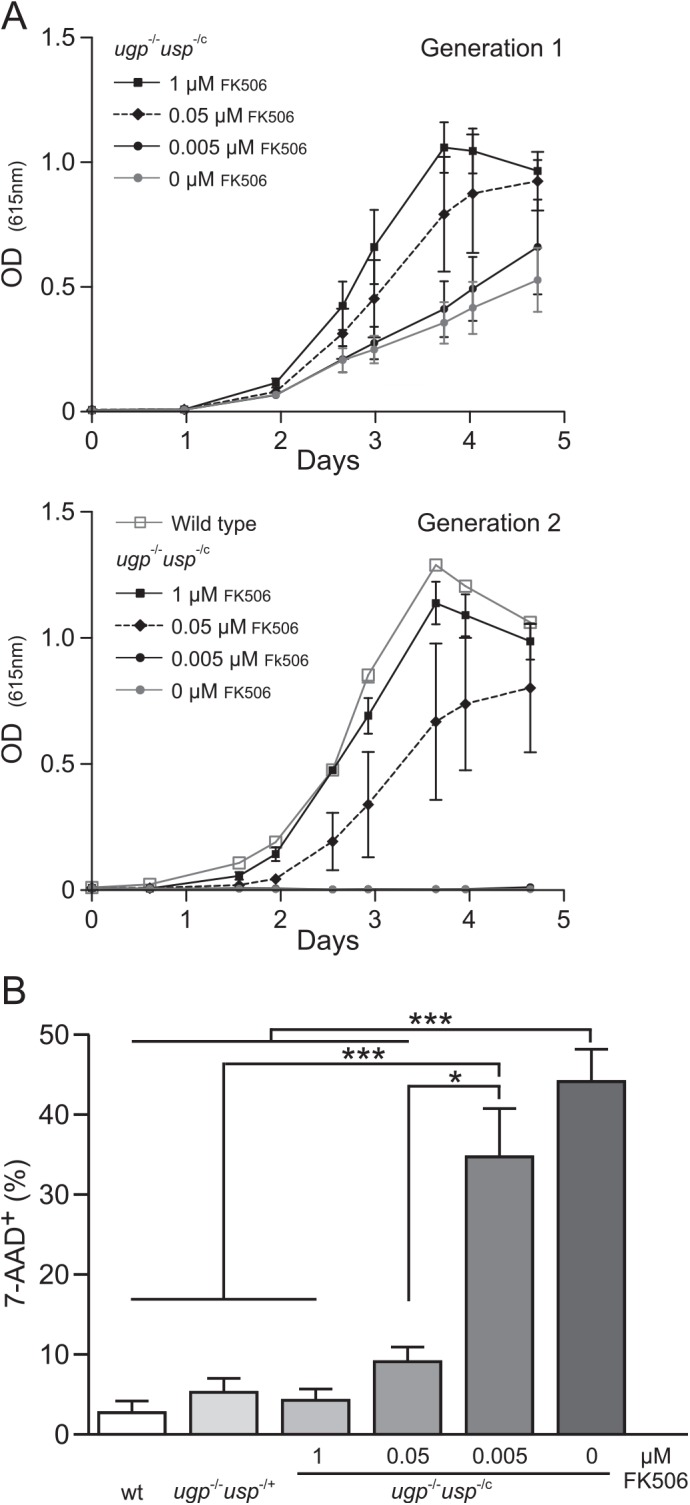
Analysis of *in vitro* growth and cell viability of *L*. *major ugp* ^-/-^ *usp* ^-/c^ mutant. (A) *In vitro* growth of wild type and *ugp* ^-/-^ *usp* ^-/c^ promastigotes grown in absence or presence of various FK506 concentrations as indicated. Medium was inoculated with 10^5^ cells (upper panel). After 3 to 4 days, parasites were transferred to fresh medium containing the indicated FK506 concentration (lower panel). Error bars indicates the standard deviation. (B) Analysis of cell viability. Parasites grown in medium containing the indicated FK506 concentration were labelled with 7-AAD and analysed by flow cytometry. Mean percentages ± SEM of dead parasites (7-AAD^+^) were calculated from four independent experiments (n = 4). Significant difference (*p < 0.01, *** p < 0.001), One-way ANOVA with Tukey post test.

Wild type parasites and the *ugp* ^-/-^ *usp* ^-/c^ mutant maintained in 1 μM FK506 were washed and transferred to medium with various FK506 concentrations at a density of 10^5^ parasites / mL. As seen in Fig 4A (upper panel), growth of the *ugp* ^-/-^ *usp* ^-/c^ mutant slowed down with decreasing FK506 concentrations. If parasites were split after 3 to 4 days (when density reached ~2–3 x 10^7^ cells/mL), the cells continued to grow with kinetics similar to wild type in the presence of 1 μM FK506, whereas no proliferation was observed if FK506 was absent or present at a concentration of 0.005 μM (Fig 4A, lower panel).

To analyse cell viability, we labelled parasites grown in medium containing different FK506 concentrations with the DNA intercalator 7-AAD and analysed them by flow cytometry. The results displayed in Fig 4B indicated that less than 5% dead cells were present in cultures of wild type *Leishmania*. The number of dead cells remained low in the *ugp* ^*-/-*^ *usp* ^*-/+*^ heterozygote or *ugp* ^-/-^ *usp* ^-/c^ cultures supplemented with 0.05μM FK506 or more. In contrast, with a FK506 concentration of 0.005μM or less, the percentage of dead cells exceeded 35%.

## Discussion

In eukaryotes, the biosynthesis of glycans, such as those creating a protective cell surface glycocalyx or cell wall, requires the activation of monosaccharides in the form of nucleotide sugars. These high-energy compounds may be formed from appropriate monosaccharides or be generated from interconversion of existing nucleotide sugars. For example, depending on the cell type, UDP-Gal, UDP-galacturonic acid, UDP-glucuronic acid, UDP-xylose and UDP-arabinose may be generated from UDP-Glc. In *Leishmania* parasites, however, the variety of nucleotide sugars synthesised is limited to UDP-Glc, UDP-Gal, UDP-GlcNAc, GDP-Man, GDP-Fuc and GDP-arabinose [23]. Previous works have shown that in this protozoan parasite, UDP-Gal is synthesised by a *de novo* pathway involving UGP [13,14] and by a salvage pathway mediated by USP [15,16]. Deficiency of UGP alone does not abolish biosynthesis of UDP-Gal and its immediate precursor UDP-Glc [13] since, as we demonstrate here, their biosynthesis is partially compensated by USP. Targeted deletion of both *UGP* and *USP* was however unsuccessful suggesting that the metabolites UDP-Glc and/or UDP-Gal are essential for *Leishmania in vitro* growth. To demonstrate the importance of the UDP-Glc/UDP-Gal biosynthesis, we therefore deleted one of the two *USP* alleles in the UGP deficient strain (*ugp* ^-/-^) and used a degron system enabling conditional degradation of the enzyme produced by the remaining *USP* allele (Fig 1). Addition of 1μM FK506 to the growth medium enabled stabilisation of the ddUSP protein product and supported restricted UDP-Glc and UDP-Gal biosynthesis, which was nonetheless sufficient to maintain parasite growth. With low FK506 concentrations, ddUSP was destabilised and the UDP-Glc/UDP-Gal biosynthesis could be reduced to minimal level leading to growth arrest and cell death, allowing us to demonstrate that UDP-Gal and/or UDP-Glc are essential metabolites for *L*. *major* promastigotes.

One of the most distinctive roles of UDP-Glc and derived nucleotide sugars is the biosynthesis of polysaccharides and glycoconjugates forming the cell surface coat. In bacteria, UGP controls the biosynthesis of the most important virulence factors and is valued as antibacterial target since it is frequently essential for pathogenicity or growth [27]. Similarly, in the trypanosomatid parasites *Trypanosoma brucei* and *Trypanosoma cruzi*, which are closely related to *Leishmania*, biosynthesis of UDP-Glc is essential for parasite growth. In these parasites, however, targeting of the UDP-glucose 4´-epimerase demonstrated that the lack of UDP-Gal, rather than its precursor UDP-Glc, is responsible for growth cessation. In both parasites, the growth phenotype was linked to alterations of the glycocalyx [19–21]. In *Leishmania*, stabilisation of the enzyme produced from a single *USP* allele with 1 μM FK506 was sufficient to maintain the steady-state UDP-Gal and UDP-Glc pools and support *in vitro* growth. The maintenance of normal UDP-Gal and UDP-Glc levels under these conditions (where *de novo* synthesis of UDP-Glc is less than 5% of wild-type) reflects a strong reduction of UDP-Gal consumption at the expense of LPG synthesis. An irreversible shutdown of LPG biosynthesis has even been observed in a heterozygote *ugp* ^-/-^ *usp* ^-/+^ clone, in adaptation to the inadequate UDP-Gal availability. In contrast, the limited UDP-Gal biosynthesis governed by a single *USP* allele was sufficient to maintain galactosylation of GIPLs. This is very similar to the situation in *T*.*cruzi* epimastigotes, where deletion of one copy of the UDP-glucose 4´-epimerase encoding gene was permissive to the synthesis of galactosylated GIPLs at the expense of the synthesis of the highly galactosylated GPI-anchored surface mucins [19]. One might assume that galactosylated GIPLs are important for *in vitro* survival and thus are retained by *L*. *major* promastigotes. However, a mutant expressing agalactosylated GIPLs due to absence of UDP-galactopyranose mutase (the enzyme that enables formation of UDP-galactofuranose necessary for addition of the first galactose residue) grew normally [24]. In fact, many *Leishmania* mutants deficient in one or several glycoconjugates have been generated and do not present major *in vitro* growth phenotypes [6]. Essentiality of the major glycoconjugates such as LPGs, PPGs, GIPLs or N-glycans can therefore be excluded.

Yet, minor components that have until now remained undetected may be crucial for parasite survival. In this regard, we know that the synthesis of GDP-Fuc is essential for *T*. *brucei* and likely also for *Leishmania* [28,29], although to date no fucose containing glycoconjugates have been described in these parasites. *Leishmania* possesses a unique GDP-Fucose biosynthetic pathway involving two bifunctional enzymes with kinase and pyrophosphorylase activities (encoded by *LmJF*16.0440 and *LmJF*16.0480) able to activate L-Fuc into GDP-Fuc. In this parasite, the classical *de novo* synthesis of GDP-Fuc from GDP-Man seems to be absent. Indeed, deletion of both fucokinase/pyrophosphorylases could only be obtained after expression of the *de novo* GDP-Fuc pathway enzymes from *Trypanosoma* [28]. In *Trypanosoma brucei*, a fucosyltransferase acting on a Gal acceptor structure has recently been characterised [30] and an orthologue is predicted in *Leishmania*. The increase of GDP-Fuc observed in the destabilised *ugp* ^-/-^ *usp* ^-/c^ mutant may thus reflect a non-utilisation of this nucleotide sugar due to the absence of acceptor structures. Thus it cannot be excluded that the phenotype observed in the *ugp* ^-/-^ *usp* ^-/c^ mutant is linked to absence of fucose containing structure(s).

As in other eukaryotes, UDP-Glc is also required in the endoplasmic reticulum (ER) for glucosylation of newly synthesised glycoproteins by the UDP-glucose:glycoprotein glucosyltransferase (UGGT); a reaction that promotes protein folding. However, in the trypanosomatids *T*. *brucei* and *T*. *cruzi*, UGGT is not essential for parasite growth in standard conditions [31,32]. Shortage of UDP-Glc in the ER might thus challenge the protein folding machinery and activates the unfolded protein response [31,33–35] but these are likely not responsible for the lethal phenotype observed under standard culture conditions.

Like other kinetoplastid flagellates, *Leishmania* parasites also need UDP-Glc in the nucleus for the synthesis of an unusual DNA base called base J (β-D-glucosyl-hydroxymethyluracil) [36]. Base J replaces at most 1% of thymidines in telomeric repeats and at transcription initiation and termination sites and was shown to regulate transcription [37]. In *Leishmania*, base J is believed to be essential since attempts to delete *JBP1*, a gene encoding one of the two thymidine hydroxylases involved in the first step of base J synthesis (formation of 5- hydroxymethyluracil), have been unsuccessful [34]. Whether glucosylation of 5-hydroxymethyluracil, is also essential for *Leishmania* growth remains to be determined. A gradual loss of residual base J upon cell division [38] could however explain the late appearance of the lethal phenotype of *L*. *major ugp* ^-/-^ *usp* ^-/c^.

Further studies are necessary to define whether UDP-Glc and/or UDP-Gal are/is the essential metabolite in *Leishmania* and to identify the downstream pathway(s) essential for parasite survival. In the trypanosomatids *T*. *brucei* and *T*. *cruzi*, both the UDP-glucose 4´-epimerase and UGP have been validated as potential therapeutic targets against trypanosomiasis [19–21,39]. However because of the high degree of similarity within the catalytic site with human UGP, specific inhibition of trypanosomatids UGPs might require the design of allosteric inhibitors [39–41]. In *Leishmania*, the existence of a salvage pathway mediated by USP, which should also be inhibited, is a further challenge for the development of specific drugs. Identification of the essential pathway(s) requiring UDP-Glc or UDP-Gal might thus provide new targets for the development of specific drugs to combat leishmaniases.

## Supporting Information

S1 Table Sequence of primers used in this study. (DOCX)

S2 Table Lipid moieties of *Leishmania major* GIPLs. (DOCX)

S1 Fig Southern blot analysis of *L*. *major ugp* ^-/-^ *usp* ^-/c^ mutant. **(**A-C) Genomic DNA from wild type (wt) and homozygous *ugp* ^-/-^ *usp* ^-/c^ mutant (Δ*ugp*::*HYG*/Δ*ugp*::*BLE*/Δ*usp*::*PAC*/*SAT-FKBP* ^*FK506i*^::*USP*) was digested with four different endonucleases, separated on agarose gel, blotted onto nylon membrane, and hybridized with digoxigenin labeled probes USP*, 5’UTR*, PAC*. The shifts of the probe labeled fragments can be assigned to the respective theoretical length in panel (D-F), indicating correct gene replacement.
(EPS)

S2 Fig Uncropped SDS-PAGE gel from Fig 2A showing equal loading. The framed area is presented in Fig 2A.
(EPS)

S3 Fig Comparison of the glycoconjugates synthesised by *ugp* ^*-/-*^ and stabilised *ugp* ^-/-^ *usp* ^-/c^ mutant. (A) Western blot of Log phase wild type, *ugp* ^*-/-*^ and stabilised *ugp* ^-/-^ *usp* ^-/c^ (medium containing 1µM FK506) promastigotes lysates probed with the anti-LPG monoclonal antibody WIC79.3 (upper panel). Loading was assessed by Coomassie staining of an identically loaded SDS-Page ran separately (lower panel). (B) Negative-ion MALDI spectra of glycosylinositolphospholipids (GIPLs) isolated from wild type, *ugp* ^*-/-*,^ *ugp* ^-/-^ *usp* ^-/+^ and stabilised *ugp* ^-/-^ *usp* ^-/c^ (grown in medium containing 1µM FK506) promastigotes. Each prominent peak is annotated with the letter a, b, c or d referring to the structure depicted under. The m/z value of prominent peaks has been indicated in the top spectrum. The lipid moiety consists of a 1-alkyl-2-*lyso*-phosphatidylinositol or 1-alkyl-2-acyl-phosphatidylinositol. The presence and length of the alkyl and acyl chains is indicated in S2 Table.
(EPS)
