# Correction: Depletion of UDP-Glucose and UDP-Galactose Using a Degron System Leads to Growth Cessation of *Leishmania major*

**DOI:** 10.1371/journal.pntd.0004372

**Published:** 2016-01-06

**Authors:** Sebastian Damerow, Carolin Hoppe, Giulia Bandini, Patricia Zarnovican, Falk F. R. Buettner, Carsten G. K. Lüder, Michael A. J. Ferguson, Françoise H. Routier

The fifth author’s name is missing an initial. The correct name is: Falk F.R. Buettner. The correct citation is: Damerow S, Hoppe C, Bandini G, Zarnovican P, Buettner FFR, Lüder CGK, et al. (2015) Depletion of UDP-Glucose and UDP-Galactose Using a Degron System Leads to Growth Cessation of Leishmania major. PLoS Negl Trop Dis 9(11): e0004205. doi:10.1371/journal.pntd.0004205
