# Supplementary material for: Depletion of UDP-Glucose and UDP-Galactose Using a Degron System Leads to Growth Cessation of Leishmania major
Source: PLoS Negl Trop Dis. 2015 Nov 3;9(11):e0004205. doi: 10.1371/journal.pntd.0004205 (PMC4631452; doi:10.1371/journal.pntd.0004205)
Supplement: S1 Table — (DOCX) [file pntd.0004205.s001.docx]

**S1 Table: Sequence of primers used in this study**

| Primer name | Sequence (5’$\boldsymbol{\to}$3’) |
| --- | --- |
| OL-PAC fw | TTCCCCCCGCCGAGCCCCTCTGCTCTCTCCTTTTCTGTCGTCACGCGGCCTTATGACCGAGTACAAGCCCACGG |
| OL-PAC rev | CATTCAACTACACTGGAACACCCACACTAGCAAGGGCCCTCTCAACAACAATCAGGCACCGGGCTTGCGGGTC |
| 5UTR_1 fw | CTGACTGAGCGGCCGCTTGCTGATGAGGGAAGGATCTGC |
| 5UTR_1 rev | AAGGCCGCGTGACGACAGAAAAGG |
| 3UTR_1 fw | TTGTTGTTGAGAGGGCCCTTGC |
| 3UTR_1 rev | CTGACTGAGCGGCCGCACAGGAGCGACCTGCGACGACG |
| 5UTR_3 fw | CTGACTGAGCGGCCGCACGGTGCTGAGGACTGCG |
| 3UTR_3 rev | CTGACTGAGCGGCCGCTGCTGCAGCTCTGGCGAGC |
| SD177 | ACTGCATATGTTGTGTGCCGCATCGTGTTG |
| SD178 | ACTGACTAGTAAGGCCGCGTGACGACAG |
| SD175 | ACTGAGATCTACGAACCCGTCCAACTC |
| USP3rev | GGCTGTCTTCTTCAGCACAAA |
| SD1 | ATGACGAACCCGTCCAACTC |
| USP1rev | CCGGAGGTAGTAGGCGAGATA |
| SD176 | ACTGCATATGATGTTTTCGTGCAGCTCC |
| SD21 | AGGCCGCGTGACGACAGAAAAGG |
| SD70 | ATGACCGAGTACAAGCCCAC |
| SD71 | TTGCGGGTCATGCACCAG |

Restriction sites are underlined
