# Supplementary material for: Depletion of UDP-Glucose and UDP-Galactose Using a Degron System Leads to Growth Cessation of Leishmania major
Source: PLoS Negl Trop Dis. 2015 Nov 3;9(11):e0004205. doi: 10.1371/journal.pntd.0004205 (PMC4631452; doi:10.1371/journal.pntd.0004205)
Supplement: S2 Table — (DOCX) [file pntd.0004205.s002.docx]

**S2 Table: Lipid moieties of *Leishmania major* GIPLs**

| Calculated m/z | Structure | Lipid moiety | Acyl chain length | Alkyl chain length |
| --- | --- | --- | --- | --- |
| 1154.6 | iM2 | 1-alkyl-2-*lyso*-PI | / | C24:0 |
| 1182.6 | iM2 | 1-alkyl-2-*lyso*-PI | / | C26:0 |
| 1252.7 | iM2* | 1-alkyl-2-acyl-PI | C12:0 | C18:0 |
| 1280.7 | iM2* | 1-alkyl-2-acyl-PI | C14:0 | C18:0 |
| 1308.7 | iM2* | 1-alkyl-2-acyl-PI | C16:0 | C18:0 |
| 1136.8 | iM2* | 1-alkyl-2-acyl-PI | C12:0 | C24:0 |
| 1364.8 | iM2* | 1-alkyl-2-acyl-PI | C14:0 | C24:0 |
| 1316.7 | GIPL-1 | 1-alkyl-2-*lyso*-PI | / | C24:0 |
| 1344.7 | GIPL-1 | 1-alkyl-2-*lyso*-PI | / | C26:0 |
| 1414.7 | GIPL-1 | 1-alkyl-2-acyl-PI | C12:0 | C18:0 |
| 1442.8 | GIPL-1 | 1-alkyl-2-acyl-PI | C14:0 | C18:0 |
| 1470.8 | GIPL-1 | 1-alkyl-2-acyl-PI | C16:0 | C18:0 |
| 1498.8 | GIPL-1 | 1-alkyl-2-acyl-PI | C12:0 | C24:0 |
| 1526.9 | GIPL-1 | 1-alkyl-2-acyl-PI | C14:0 | C24:0 |
| 1478.7 | GIPL-2 | 1-alkyl-2-*lyso*-PI | / | C24:0 |
| 1576.8 | GIPL-2 | 1-alkyl-2-acyl-PI | C12:0 | C18:0 |
| 1604.8 | GIPL-2 | 1-alkyl-2-acyl-PI | C14:0 | C18:0 |
| 1660.9 | GIPL-2 | 1-alkyl-2-acyl-PI | C12:0 | C24:0 |
| 1688.9 | GIPL-2 | 1-alkyl-2-acyl-PI | C14:0 | C24:0 |
| 1640.8 | GIPL-3 | 1-alkyl-2-*lyso*-PI | / | C24:0 |
| 1738.9 | GIPL-3 | 1-alkyl-2-acyl-PI | C12:0 | C18:0 |
| 1766.9 | GIPL-3 | 1-alkyl-2-acyl-PI | C14:0 | C18:0 |
| 1822.9 | GIPL-3 | 1-alkyl-2-acyl-PI | C12:0 | C24:0 |
| 1851.0 | GIPL-3 | 1-alkyl-2-acyl-PI | C14:0 | C24:0 |
| 1418.6^a^ | LPG precursor* | 1-alkyl-2-*lyso*-PI | / | C24:0 |
| 1446.6^a^ | LPG precursor* | 1-alkyl-2-*lyso*-PI | / | C26:0 |

* Not present in wild type

^a^ [M-2H +Na^+^]^-^
